# Association of Work Environment With Missed and Rushed Care Tasks Among Care Aides in Nursing Homes

**DOI:** 10.1001/jamanetworkopen.2019.20092

**Published:** 2020-01-29

**Authors:** Yuting Song, Matthias Hoben, Peter Norton, Carole A. Estabrooks

**Affiliations:** 1 Faculty of Nursing, University of Alberta, Edmonton, Alberta, Canada; 2 Department of Family Medicine, University of Calgary, Calgary, Alberta, Canada

## Abstract

**Importance:**

In Canada, approximately 81% of residents of nursing homes live with mild to severe cognitive impairment. Care needs of this population are increasingly complex, but resources, such as staffing, for nursing homes continue to be limited. Staff risk missing or rushing care tasks and interfering with quality of care and life.

**Objective:**

To assess the association of work environment with missing and rushing essential care tasks in nursing homes.

**Design, Setting, and Participants:**

This cross-sectional study used survey data collected from a random sample of 93 urban nursing homes in Western Canada, stratified by health region, owner-operator model, and facility size, between May and December 2017. All 5411 eligible care aides were invited to participate, and 4016 care aides agreed and completed structured, computer-assisted interviews in person. Analyses were conducted from July 4, 2018, to February 27, 2019.

**Main Outcomes and Measures:**

Self-reported number of essential care tasks missed (range, 0-8) or rushed (range, 0-7) in the most recent shift. Two-level random intercept hurdle regressions controlled for care aide, care unit, and nursing home characteristics.

**Results:**

Of 4016 care aides, 2757 (68.7%) were 40 years or older, 3574 (89.1%) were women, and 1353 (66.3%) spoke English as an additional language. For their most recent shift, 2306 care aides (57.4%) reported missing at least 1 essential care task and 2628 care aides (65.4%) reported rushing at least 1 essential care task. Care aides on units with more favorable work environments (eg, more effective leadership, better work culture, higher levels of buffering resources) were less likely to miss any care tasks (odds ratio, 1.59; 95% CI, 1.34-1.90; *P* < .001) and less likely to rush any care task (odds ratio, 1.66; 95% CI, 1.38-1.99; *P* < .001).

**Conclusions and Relevance:**

This study found that rates of missed and rushed essential care in Canadian nursing homes were high and were higher in units with less favorable work environments. This finding suggests that work environment should be added to the list of modifiable factors associated with improving nursing home care, as it may be an important pathway for improving quality of care. Further research is needed to understand associations of missed and rushed care and of improving work environments with outcomes among residents of nursing homes.

## Key Points

**Question:**

Is work environment associated with noncompletion and rushing of essential care tasks by nursing home care aides?

**Findings:**

In this cross-sectional study of 4016 care aides in 93 Canadian nursing homes, care aides in homes with more favorable work environments were 59% less likely to miss care tasks and 66% less likely to rush care tasks.

**Meaning:**

These findings suggest that fewer essential care tasks are missed or rushed in nursing homes with more favorable work environments.

## Teaser

This cross-sectional study examines the association of care aides missing or rushing essential care tasks with work environments in nursing homes in Canada.

## Introduction

Nursing homes care for people with complex medical and social care needs. More than 80% of direct care is performed by care aides (also called *nursing assistants*).^1^ Despite increasingly complex care needs of residents, nursing home funding remains limited,^2^ while workloads of care aides increase and become more complex. Care aides may rush and miss care tasks to meet tight schedules and task lists,^3^ although those tasks are essential to both quality of care and quality of life. Emerging research suggests that care aides frequently leave essential care undone^4,5^ and rush essential care.^6^

Both quality of care and quality of life for residents likely diminish with missed and rushed care, but it is not fully understood why missed and rushed care happens. That knowledge is critical for interventions to reduce it. Kaplan et al^7^ identified optimized organizational context (ie, work environment) as important to successful interventions for quality improvement. In acute care, better organizational context is associated with less missed care.^8,9,10,11^ However, to our knowledge, there are no data for nursing homes.

We examined how modifiable elements of organizational context are associated with missed and rushed care by care aides in nursing homes to inform the design of interventions that could reduce missed and rushed care. We hypothesized that care aides working on care units with more favorable organizational context would report fewer missed and rushed essential care tasks than those working on care units with less favorable context.

## Methods

### Study Design

This study used cross-sectional survey data from the Translating Research in Elder Care (TREC) research program,^12^ which is a multilevel, longitudinal program of applied health services research to improve quality of care and quality of life for residents of nursing home and quality of work life for the staff who care for them. Since 2007, TREC has collected data from a cohort of 93 nursing homes across Western Canada. Variables used in this study were initially developed in 2012, piloted during data collection from 2014 to 2015, and first comprehensively collected from May to December 2017. This study is reported following Strengthening the Reporting of Observational Studies in Epidemiology (STROBE) reporting guideline.

### Setting

Canada’s health regions oversee most aspects of nursing homes, including quality of care and nursing home policies, with exceptions in some regions for for-profit operators.^13^ Health regions vary in oversight.^13^ Study facilities for TREC were randomly selected from lists stratified by health region (ie, British Columbia Fraser Health Authority, British Columbia Interior Health Authority, Alberta Health Edmonton Zone, Alberta Health Calgary Zone, or Winnipeg Regional Health Authority), owner-operator model (ie, public nonprofit, voluntary nonprofit, or private for-profit), and facility size (ie, small, defined as <80 beds; medium, 80-120 beds; or large, >120 beds). Specific sampling and data collection approaches are described in detail elsewhere.^12^ Care units (also known as *clinical microsystems*^14,15^) in this study were physical locations in nursing homes with a unit manager, a nurse overseeing shift-by-shift care, and dedicated teams of regular staff providing direct care. These clinical microsystems are essential foci for improvement programs.

### Ethics

This study was approved by the University of Alberta research ethics board. For original TREC survey data collection, participating organizations gave operational approvals. Using multiple strategies, we informed potential survey participants about this study. Written informed consent was obtained from participants.

### Participants

Care aides were eligible to participate if they had worked in a study facility for longer than 3 months, could identify a unit where they worked for at least 50% of their time during the data collection period, and worked on that unit for 6 or more shifts in the past month. Trained TREC data collectors went to each participating nursing home and invited all eligible care aides to participate.^12^ Care aides who agreed to participate completed computer-assisted, structured interviews in person^16^ between May and December 2017.

Only care units with responses from at least 8 care aides were included in this study. This criterion reflects our finding that stable estimates of organizational context at the unit level are achieved when 8 or more individual responses are aggregated.^15^

### Measures

#### Independent Variable

Our independent variable was organizational context at the unit level, measured by the Alberta Context Tool (ACT). This validated instrument measures 10 modifiable elements of organizational context: leadership, culture, evaluation, formal interactions, informal interactions, structural and electronic resources, social capital, organizational slack in use of time, organizational slack in use of staff, and organizational slack in use of space.^17^ eTable 1 in the Supplement summarizes the psychometric properties of ACT. Each element has 2 to 9 items. Each element was aggregated within respondents by the mean or the count of items (Table 1). We used care aide responses to create unit-level scores for context because care aides are the only sufficiently large workforce group in nursing homes to support aggregate scores. Care aides also provide 80% or more of direct resident care^1^ and are most familiar with residents and conditions of work and resident life. They are well positioned to provide context scores that are closest to resident experiences. We aggregated to the unit level the 10 ACT elements from each care aide surveyed. Then, drawing on Milligan and Cooper’s clustering analysis approach^18^ (n of predetermined clusters = 2), we determined whether a unit had a more or less favorable organizational context.^19^

**Table 1.** Dependent, Independent, and Control Variables Measured

Variable	Description	No. of Items	Scoring
**Dependent **			
Missed care	Self-reported care tasks (ie, taking residents for a walk, talking with residents, performing mouth care, toileting, bathing residents, feeding residents, dressing residents, and preparing residents for sleep) that were left undone during respondent’s most recent shift, No.	8	Count: range, 0-8
Rushed care	Self-reported care tasks (ie, talking with residents, performing mouth care, toileting, bathing residents, feeding residents, dressing residents, and preparing residents for sleep) that were rushed during respondent’s most recent shift, No.	7	Count: range, 0-7
**Independent **			
Organizational context	Respondent’s perception of work environment of the care unit, measured with the Alberta Context Tool	1	Binary: more favorable vs less favorable
Leadership	Actions of formal leaders in an organization (unit) to influence changes and excellence in practice; items generally reflect emotionally intelligent leadership	6	Continuous: mean of items on a 5-point Likert scale in which 1 = strongly disagree and 5 = strongly agree
Culture	The way that things are done in the organizations and work units; items generally reflect a supportive work culture	6	Continuous: mean of items on a 5-point Likert scale in which 1 = strongly disagree and 5 = strongly agree
Evaluation	The process of using data to assess group or team performance and to achieve outcomes in organizations or units	6	Continuous: mean of items on a 5-point Likert scale in which 1 = strongly disagree and 5 = strongly agree
Formal interaction	Formal exchanges between individuals working within an organization or unit through scheduled activities that can promote the transfer of knowledge	4	Continuous: mean of items on a 5-point frequency Likert scale in which 1 = never and 5 = almost always; recoded to 0 = no interaction, 1 = interaction and a recount of recoded items was taken
Informal interaction	Informal exchanges among individuals working within an organization or unit that can promote the transfer of knowledge	9	Count: 5-point Likert frequency scale in which 1 = never and 5 = almost always; recoded to 0 = no interaction, 1 = interaction and a count of recoded items was taken
Structural and electronic resources	Structural elements of an organization or unit that facilitate the ability to assess and use knowledge	7	Count: 5-point Likert frequency scale in which 1 = never and 5 = almost always; recoded to 0 = no interaction, 1 = interaction and a count of recoded items was taken
Social capital	Stock of active connections among people, including 3 types of connections: bonding, bridging, and linking	6	Continuous: mean of items on a 5-point Likert scale in which 1 = strongly disagree and 5 = strongly agree
Organizational slack in use of staff	Cushion of actual or potential staff resources that allows an organization or unit to adapt successfully to internal pressures for adjustments or to external pressures for changes	3	Continuous: mean of items on a 5-point Likert scale in which 1 = strongly disagree and 5 = strongly agree
Organizational slack in use of space	Cushion of actual or potential space resources that allows an organization or unit to adapt successfully to internal pressures for adjustments or to external pressures for changes	2	Continuous: mean of items on a 5-point Likert scale in which 1 = strongly disagree and 5 = strongly agree
Organizational slack in use of time	Cushion of actual or potential time resources that allows an organization or unit to adapt successfully to internal pressures for adjustments or to external pressures for changes	4	Continuous: mean of items on a 5-point Likert scale in which 1 = strongly disagree and 5 = strongly agree
**Control**			
Age	Respondent’s age, y	1	Categorical: <20, 20-29, 30-39, 40-49, 50-59, or ≥60
Sex	Respondent’s sex	1	Binary: women or men
Education	Care aide certificate obtained	1	Binary: yes or no
English as first language	English is respondent’s first language	1	Binary: yes or no
Shift worked most often	Shift the respondent works most often	1	Categorical: day, evening, or night
Experience on current unit	Total time that the respondent has worked on their current unit, y	1	Continuous
Responsive behaviors from residents	Responsive behaviors (eg, yelling, biting, sexual touching) that respondent experienced from residents in their most recent 5 shifts, No.	1	Count: range, 0-6
Job satisfaction	Respondent satisfaction with their current job, measured with the Michigan Organizational Assessment Questionnaire Job Satisfaction Subscale.	3	Continuous: mean of items on a 5-point Likert scale in which 1 = strongly disagree and 5 = strongly agree
Burnout	Measured with the Maslach Burnout Inventory 9-item short form		
Emotional exhaustion	Respondent feels emotionally exhausted or strained	3	Continuous: mean of 7-point Likert scale in which 0 = never and 6 = daily
Cynicism	Respondent feels cynical or that their work does not contribute to anything	3	Continuous: mean of 7-point Likert scale in which 0 = never and 6 = daily
Efficacy	Respondent feels their work is meaningful or has a sense of accomplishment	3	Continuous: mean of 7-point Likert scale in which 0 = never and 6 = daily
Health status	Measured with the 8-item Short Form Survey		
Physical health	Respondent’s perception of their own physical health in the most recent 4 wk	8	Continuous: scoring of items on 5- or 6-point Likert scales based on scale developers’ algorithm
Mental health	Respondent’s perception of their own mental health in the most recent 4 wk	8	
**Unit Level**			
Type	Type of care unit on which respondent works ≥50% of their time	1	Categorical: general nursing home, secure dementia, mental health, combined nursing home and dementia
Staffing level	Time worked by registered nurses, licensed practical nurses, and care aides per resident per day, h	1	Continuous
% of care aide staffing out of total unit staffing	Time worked by care aides per resident per d out of time worked by registered nurses, licensed practical nurses, and care aides per resident per d, %	1	Continuous: range, 0-100
**Nursing Home Level **			
Owner-operator model	Ownership model of the nursing home	1	Categorical: public nonprofit, voluntary nonprofit, private for-profit
Size	Beds in the nursing home, No.	1	Categorical: small, ≤79; medium, 80-120; large, >120
Health region	Health region that regulates the nursing home	1	Categorical: BC Fraser Health Authority, BC Interior Health Authority, Alberta Health Edmonton Zone, Alberta Health Calgary Zone, Winnipeg Regional Health Authority

Abbreviation: BC, British Columbia.

#### Outcomes

Our dependent variables were missed care and rushed care at the individual care aide level. Missed care and rushed care items were developed iteratively by engaging care aides in developing previous TREC surveys.^4^ Items followed the form, *on your last shift, did you leave mouth care for residents undone because you did not have enough time?* Care aides were asked yes or no for each missed and rushed task item. Yes responses were counted and summed separately for missed care and rushed care.

#### Covariates

For multivariable analysis, we adjusted for covariates related to care aide, unit, and nursing home characteristics (Table 1). Inclusion of these variables in final models was informed by previous research on missed care in acute settings and nursing homes.^4,6,8,20^ Care aides self-reported their demographic and work characteristics.^12^ Unit and facility characteristics were collected in short structured interviews with unit and facility managers. For missing data, we used listwise deletion. Less than 0.2% of data were missing for all variables, completely at random (Little missing completely at random test,^21^ *P* = .84). eTable 2 in the Supplement shows characteristics of care aides with complete vs incomplete data.

### Statistical Analysis

Analyses were conducted from July 4, 2018, to February 27, 2019, using SAS statistical software version 9.4 (SAS Institute). For descriptive analysis, variable categories were care aide demographic characteristics, care aide work characteristics, unit and facility characteristics, and health region. We calculated means and SDs for continuous variables and frequency counts and proportions for categorical and binary variables. We summed the number of times that care aides answered yes to missing or rushing each care task and calculated percentage of occurrence in the whole sample. We then ranked missed and rushed care tasks from highest to lowest percentage of occurrence in the whole sample. To inform inclusion of variables in subsequent multivariable analysis, we drew on previous literature on missed and rushed care in short- and long-term care settings. Multicollinearity was assessed, and 2 variables (ie, hours worked in 2 weeks and time worked as a care aide) were removed before multivariable analysis.

For regression analyses, we ran 2 sets of analyses, one using ACT as a binary variable and the other with the 10 ACT elements individually. Hurdle Poisson regression models were used for both analyses. They account for excessive numbers of 0s using 2 components: logistic and Poisson regression models. The logistic model recodes the dependent variable (ie, missed care or rushed care) as a binary variable: 0 or 1. It models probability when the dependent variable is 0. The Poisson regression model only uses data with a non-0 dependent variable: 1 to 8 for missed care and 1 to 7 for rushed care. Odds ratios (ORs) with 95% CIs were generated for the logistic model. Relative rates (RRs) with 95% CIs were generated for the Poisson model. To control for the clustering effect of individual care aides within units, we added a random intercept parameter to each component. *P* values were 2-tailed, and statistical significance was set at *P* < .01.

## Results

Of 5411 eligible care aides in 312 care units in 93 nursing homes, 4016 care aides (74.2%) responded to the survey invitation. Data to assess nonresponder characteristics were not available because data on nursing home care aides in Canada are not kept systematically.^13^ Characteristics of included care aides are presented in Table 2. Care aides were predominantly women (3574 care aides [89.1%]) 40 years or older (2757 care aides [68.7%]) who spoke English as an additional language (1353 care aides [66.3%]) (Table 2). Characteristics of care units and nursing homes are presented in Table 3.

**Table 2.** Care Aide Characteristics

Variable	No. (%) (N = 4016)
Age, y	
<29	396 (9.9)
30-39	863 (21.5)
40-49	1275 (31.7)
50-59	1046 (26.0)
≥60	436 (10.9)
Women	3574 (89.1)
Health Care Aide certificate	3758 (93.6)
English as first language	1353 (33.7)
Shift worked most often	
Day	1968 (49.0)
Evening	1578 (39.3)
Night	470 (11.7)
Experience, mean (SD)	
On current unit, y	5.8 (5.9)
Responsive behaviors by residents, No.	3.3 (1.7)
Job satisfaction score, mean (SD)^a^	4.2 (0.6)
Burnout score, mean (SD)^b^	
Emotional exhaustion	2.6 (1.7)
Cynicism	2.7 (1.6)
Efficacy	5.4 (0.8)
Health score, mean (SD)^c^	
Physical	49.1 (8.2)
Mental	51.9 (8.4)

^a^ Measured with the Michigan Organizational Assessment Questionnaire Job Satisfaction Subscale.

^b^ Measured with the Maslach Burnout Inventory 9-item short form.

^c^ Measured with the 8-item Short Form Survey.

**Table 3.** Unit and Nursing Home Characteristics

Characteristic	No. (%)
**Unit Level**	
No.	312 (100)
Organizational context	
More favorable	167 (53.5)
Less favorable	145 (46.5)
Type	
General nursing home	208 (66.7)
Secure dementia	45 (14.4)
Secure mental health or psychiatric	3 (1.0)
Nonsecure dementia	14 (4.5)
Other	42 (13.5)
Staffing level, mean (SD), h/resident/d	2.7 (0.9)
Care aide staffing, mean (SD), %	70.0 (11.3)
**Nursing Home Level**	
No.	93 (100)
Owner-operator model	
Public nonprofit	19 (20.4)
Private for-profit	40 (43.0)
Voluntary nonprofit	34 (36.6)
Size	
Small	20 (21.5)
Medium	36 (38.7)
Large	37 (39.8)
Health region	
BC Fraser Health Authority	26 (28.0)
BC Interior Health Authority	16 (17.2)
Alberta Health Edmonton Zone	20 (21.5)
Alberta Health Calgary Zone	15 (16.1)
Winnipeg Regional Health Authority	16 (17.2)
Units within nursing home, mean (SD), No.	3.4 (1.9)

Abbreviation: BC, British Columbia.

Missing and rushing care tasks were common in nursing homes. For their most recent shift, 2306 care aides (57.4%) reported missing at least 1 care task and 2628 care aides (65.4%) reported rushing at least 1 care task (Table 4). The most frequently missed task was taking residents for a walk, which 1492 care aides (37.2%) reported missing. The most frequently rushed task was talking with residents, which 1977 care aides (49.2%) reported rushing. Performing mouth care was missed by 567 care aides (14.1%) and rushed by 1580 care aides (39.3%). Other care tasks, such as toileting, preparing residents for sleep, bathing residents, feeding residents, and dressing residents, were each missed by less than 10% of care aides but were each rushed by more than 30% of care aides (Table 4).

**Table 4.** Missed and Rushed Care Tasks Reported

Care Task	Care Aides, No. (%) (N = 4016)
Missed	2306 (57.4)
Taking residents for a walk	1492 (37.2)
Talking with residents	1315 (32.7)
Performing mouth care	567 (14.1)
Toileting	383 (9.5)
Preparing residents for sleep	292 (7.3)
Bathing	283 (7.1)
Feeding	249 (6.2)
Dressing	213 (5.3)
Rushed	2628 (65.4)
Talking with residents	1977 (49.2)
Dressing	1797 (44.7)
Toileting	1700 (42.3)
Feeding residents	1598 (39.8)
Performing mouth care	1580 (39.3)
Bathing residents	1528 (38.0)
Preparing for sleep	1221 (30.4)

Compared with care aides who worked on units with less favorable organizational context, care aides who worked on units with more favorable organizational context were 59% less likely to miss any care task (OR, 1.59; 95% CI, 1.34-1.90; *P* < .001) and 66% less likely to rush any care task (OR, 1.66; 95% CI, 1.38-1.99; *P* < .001) (Table 5). Among care aides who reported rushing at least 1 task, those working on units with more favorable organizational context rushed 7% fewer care tasks (RR, 0.93; 95% CI, 0.88-0.98; *P* = .007) than care aides on units with less favorable organizational context (eTable 3 and eTable 4 in the Supplement).

**Table 5.** Multivariable Analyses of Organizational Context With Missed Care and Rushed Care^a^

Organizational Context	Missed Care						Rushed Care					
	Logistic Component^b^			Poisson Component^c^			Logistic Component^b^			Poisson Component^c^		
	OR (95% CI)	Estimate	*P* Value	RR (95% CI)	Estimate	*P* Value	OR (95% CI)	Estimate	*P* Value	RR (95% CI)	Estimate	*P* Value
Less favorable	1 [Reference]	NA	NA	1 [Reference]	NA	NA	1 [Reference]	NA	NA	1 [Reference]	NA	NA
More favorable	1.59 (1.34-1.90)	0.47	<.001	0.92 (0.84-1.01)	−0.08	.08	1.66 (1.38-1.99)	0.50	<.001	0.93 (0.88-0.98)	−0.07	.007
Random intercept for unit clustering	1.08 (1.01-1.17)	0.08	.03	1.04 (1.01-1.06)	0.03	.001	1.06 (0.97-1.15)	0.06	.19	1.01 (1.00-1.02)	0.009	.009

Abbreviations: OR, odds ratio; NA, not applicable; RR, relative rate.

^a^ Adjusted for age, sex, education, language, shift worked most often, time worked on current unit, experience of responsive behaviors from residents, job satisfaction, burnout, physical health, mental health, unit type, unit staffing level, percentage of care aide staffing hours out of total staffing hours, owner-operator model, nursing home size, and health region.

^b^ Models the likelihood of 0 missed or rushed care tasks.

^c^ Compares numbers of missed or rushed care tasks among individuals with at least 1 missed or rushed care task.

To understand which specific elements of organizational context were associated with missed and rushed care in our sample, we conducted further analyses with the 10 ACT elements. Missed care was associated with 4 elements: culture, organizational slack in use of staffing or use of time, and social capital (eTable 5 in the Supplement). With a 1-unit increase in care unit culture, care aides were 62% more likely to miss any care task (OR, 0.38; 95% CI, 0.19-0.73; *P* = .004). However, with a 1-unit increase in organizational slack in use of staff, care aides were 65% less likely to miss any care task (OR, 1.65; 95% CI, 1.32-2.05; *P* < .001). For organizational slack in use of time, care aides were 103% less likely to miss any care tasks (OR, 2.03; 95% CI, 1.44-2.86; *P* < .001). Among care aides who reported missing at least 1 care task, a 1-unit increase in social capital was associated with missing 49% fewer care tasks (RR, 0.51; 95% CI, 0.36-0.70; *P* < .001), and a 1-unit increase of organizational slack in use of staff was associated with missing 20% fewer care tasks (RR, 0.80; 95% CI, 0.71-0.90; *P* < .001) (eTable 5 in the Supplement).

Rushed care was associated with 2 elements: organizational slack in use of staff and culture. With a 1-unit increase of organizational slack in use of staff on care units, care aides were 91% less likely to rush any care task (OR, 1.91; 95% CI, 1.51-2.42; *P* < .001). However, care aides who reported rushing at least 1 task rushed 34% more care tasks with a 1-unit increase in culture (RR, 1.34, 95% CI, 1.09-1.65; *P* = .006) (eTable 6 in the Supplement).

## Discussion

In this cross-sectional study, high proportions of nursing home care aides reported missing and rushing essential care tasks—tasks that are important to quality of care and quality of resident life. Excessive staff busyness has been identified as a significant daily challenge in nursing homes in Europe,^20^ Canada,^4^ Japan,^22^ and Australia.^23^ Care aides’ work is frequently interrupted, with only 1- to 3-minute uninterrupted intervals provided for approximately half of care tasks, including feeding and bathing.^6^ In Switzerland, nurses and care aides reported missing 46% of activities of daily living at least once in the previous 7 days.^20^ In Japan, care workers have described only having time for minimum care (eg, bathing, changing diapers) and none for extra care, such as taking residents for a walk.^22^

Research from acute settings supports that missing or rushing essential care interferes with quality and safety.^8,24^ Missed care explained 40% of variation in quality ratings of US-based acute care hospitals^25^ and 9.2% of variance in patient falls.^9^ In US and European acute care studies, missed care was associated with increased nosocomial infections, pressure ulcers, patient dissatisfaction, medication errors, readmission to hospitals, critical incidents, compromised patient safety, and increased mortality.^8,9,26^ A 2018 study across 9 European countries^27^ found that every 10% increase in missed care by nurses was associated with a 16% increase in odds of 30-day postoperative mortality. That study^27^ also found that missed care was associated with mediating the association of nurse staffing level with postoperative mortality rate. From these findings, Ball and Griffiths^28^ concluded that missed nursing care (ie, errors of omission) should be a key patient safety measure in hospitals alongside errors of commission. These reports suggest value in measuring and addressing missed care in nursing homes.

Almost no research is available on rushed care, to our knowledge. However, evidence is emerging on the benefits of so-called slow care, the opposite of rushed care, for residents of nursing homes, especially those living with dementia.^29^ Staff who can give residents the time they need facilitate a sense of coherence and foster dignity.^29,30^ Unrushed care by staff has potential to decrease responsive behaviors of residents.^31^ These studies strongly suggest that there are potential harms for residents when staff rush care tasks.

The 2 most frequently missed and rushed care tasks for nursing home care aides in our sample were walking and talking with residents. Talking is directly associated with preventing loneliness and boredom and with encouraging social engagement and creation of meaning. Walking is directly associated with mobility, a serious challenge in nursing homes.^32^ Immobility is associated with multiple adverse health and quality of life outcomes (eg, fecal incontinence, pressure ulcers, skin tears).^32,33,34^ Both talking and walking with residents are associated with care quality and quality of life.

In our study, care aides working on care units with more favorable organizational context were less likely to miss or rush care tasks. Research has documented that modifiable features of organizational context (eg, leadership, culture, team communication) are associated with resident outcomes^35,36,37,38,39^ and that these features are interrelated.^40,41,42^ Each element of organizational context is modifiable and offers intervention possibilities. However, we propose that nursing home managers and researchers use organizational context as an omnibus construct, modifying multiple elements simultaneously instead of targeting single elements as they develop quality improvement interventions for resident outcomes. We adopted a validated measure of organizational context that draws on a conceptual framework^43^ and is operationalized through a rigorous process of cluster analysis^18^ with the 10 modifiable ACT elements. Our findings may provide potential directions for nursing home managers to reduce missed and rushed care by care aides through improved local organizational context.

We found that units for which care aides perceived more organizational slack in use of staff and use of time had lower likelihoods or numbers of missed and rushed essential care tasks. Research has reported mixed evidence on the association of nursing staffing and resident outcomes, such as 2 systematic reviews in nursing homes that concluded that the evidence was inconsistent—higher staffing levels were associated with both better and worse resident outcomes.^44,45^ However, staff perceptions of staffing level may differ from actual levels. Perceptions are affected by care unit composition, staff composition, and resident composition.^46^ In our study, we identified variation in care aide perceptions of staffing and time across care units, although staffing levels for nursing homes (eg, number of care hours per resident day) are essentially constant across Western Canadian jurisdictions.^2,47,48^ While funding for nursing homes continues to be limited, our finding suggests opportunities for nursing home managers to improve care aides’ perceptions of staffing. Focus on organizational context and its various elements may reduce essential care tasks missed or rushed by care aides.

We found that care aides on units with better work environment culture had higher likelihood or number of missed and rushed essential care tasks, opposite to our hypothesis. We do not yet understand why, but we urge further research on this association.

We found that care aides on units with higher levels of social capital (eg, active connections through information sharing) missed fewer care tasks. Existing evidence is mixed on the association of work environment social capital with resident outcomes. Leonard et al^49^ have argued that effective communication is key to safe care; however, a randomized clinical trial by Colón-Emeric et al^50^ teaching nursing home staff to improve connections with coworkers reported improved staff communication but not an increased number of fall risk reduction activities. Our finding offers evidence for the association of social capital with less frequently missed care, providing a potential mechanism for the association of social capital in care units with resident outcomes.

### Strengths and Limitations

Our study has some strengths, including that we controlled for the clustering effect of care aides within care units in our analyses^14^ and we used a large stratified random sample of nursing homes.^12^ Data collection used a rigorous in-person structured interview process with real-time data quality assessments. We included robust data and findings on both missed and rushed care, generating a more complete portrait of these phenomena in nursing homes than previous research has generated by studying only missed or rushed care.^6,20^

Our study also has some limitations. We used survey data, which may be susceptible to self-report biases, although recall bias was reduced by asking aides to report on their most recent shift. The potential for misreporting, such as underreporting of missed and rushed care tasks, was reduced by our interview structure and data quality assessments. The study may have bias from omitted variables because we did not control for resident characteristics (eg, cognitive status, responsive behaviors) associated with missed and rushed care.^5^ We reduced bias by controlling for multiple proxy variables for these characteristics, such as unit type (eg, general long-term care, dementia care units) and the experiences of care aides of residents’ responsive behaviors. Also, the continuous part of the Hurdle Poisson regression models assumes that categories are equidistant. In addition, we did not include sampling weights in our models, as we were not interested in exact estimates of true population values but were interested in possible associations. For this purpose, sampling weights were less relevant.^51^ Furthermore, data were obtained from a stratified random sample of 93 of 524 facilities in the 3 provinces in Western Canada; thus, caution should be taken with wider generalizations.

## Conclusions

This study’s findings suggest that rates of missed and rushed essential care are high, which may put residents of nursing homes at risk of adverse health outcomes and decreased quality of life. Researchers, policy makers, and health care system and nursing home managers should consider adding work environment to a list of modifiable factors to improve care and offer new intervention pathways for improving care quality.

## Notes

Supplement. **eTable 1.** Psychometric Properties of the Alberta Context Tool **eTable 2.** Characteristics of Care Aides With Complete Data and Those With Incomplete Data **eTable 3.** Multivariable Analyses of Organizational Context With Missed Care **eTable 4.** Multivariable Analyses of Organizational Context With Rushed Care **eTable 5.** Multivariable Analyses of 10 Elements of Organizational Context With Missed Care **eTable 6.** Multivariable Analyses of 10 Elements of Organizational Context With Rushed Care
