# Error in Abstract and Results

**DOI:** 10.1001/jamanetworkopen.2020.4547

**Published:** 2020-04-03

In the Original Investigation titled “Association of Work Environment With Missed and Rushed Care Tasks Among Care Aides in Nursing Homes,”^1^ published January 29, 2020, there was an error in the Abstract and in the Results. The number of care aides who spoke English as an additional language should have been 2663 care aides. This article has been corrected.^1^
