# Supplementary material for: Association of Work Environment With Missed and Rushed Care Tasks Among Care Aides in Nursing Homes
Source: JAMA Netw Open. 2020 Jan 29;3(1):e1920092. doi: 10.1001/jamanetworkopen.2019.20092 (PMC6991287; doi:10.1001/jamanetworkopen.2019.20092)
Supplement: Supplement. — eTable 1. Psychometric Properties of the Alberta Context Tool eTable 2. Characteristics of Care Aides With Complete Data and Those With Incomplete Data eTable 3. Multivariable Analyses of Organizational Context With Missed Care eTable 4. Multivariable Analyses of Organizational Context With Rushed Care eTable 5. Multivariable Analyses of 10 Elements of Organizational Context With Missed Care eTable 6. Multivariable Analyses of 10 Elements of Organizational Context With Rushed Care [file jamanetwopen-3-e1920092-s001.pdf]

Supplementary Online Content

Song Y, Hoben M, Norton P, Estabrooks CA. Association of work environment with missed and rushed care tasks among care aides in nursing homes. *JAMA Netw Open*. 2020;3(1):e1920092. doi:10.1001/jamanetworkopen.2019.20092

eTable 1. Psychometric Properties of the Alberta Context Tool

eTable 2. Characteristics of Care Aides With Complete Data and Those With Incomplete Data

eTable 3. Multivariable Analyses of Organizational Context With Missed Care

eTable 4. Multivariable Analyses of Organizational Context With Rushed Care

eTable 5. Multivariable Analyses of 10 Elements of Organizational Context With Missed Care

eTable 6. Multivariable Analyses of 10 Elements of Organizational Context With Rushed Care

This supplementary material has been provided by the authors to give readers additional information about their work.

© 2020 Song Y et al. *JAMA Network Open.*

eTable 1. Psychometric Properties of the Alberta Context Tool

**Reliability**	Result
Internal consistency	The Cronbach's alpha for 8 of the 10 elements exceeded 0.7, which is a commonly accepted standard.
**Validity**	
Internal structure	Confirmatory factor analyses demonstrated that the overall pattern of the data tested was consistent with the structure hypothesized in the development of ACT.
Convergent validity	Bivariate associations with instrumental research use (which the ACT was designed to predict) showed that more positive contextual were statistically significant for 8 of the 10 ACT elements.

© 2020 Song Y et al. *JAMA Network Open.*

eTable 2. Characteristics of Care Aides With Complete Data and Those With Incomplete Data

Variable	Care aides with complete data n (%)	Care aides with incomplete data n (%)	Total n (%)
**Number of care aides**	3957 (98.53)	59 (1.47)	4016 (100)
**Age, y**			
<29	391 (9.88)	5 (8.47)	396 (9.86)
30–39	852 (21.5)	11 (18.6)	863 (21.5)
40–49	1251 (31.6)	24 (40.7)	1275 (31.7)
50–59	1030 (26.0)	16 (27.1)	1046 (26.0)
>60	433 (10.9)	3 (5.08)	436 (10.9)
**Female**	3529 (89.2)	45 (83.3)	3574 (89.1)
**Health Care Aide certificate**	3702 (93.6)	56 (96.6)	3758 (93.6)
**English as first language**	1327 (33.5)	26 (44.8)	1353 (33.7)
**Shift worked most often**			
Day	1940 (49.0)	28 (47.5)	1968 (49.0)
Evening	1556 (39.3)	22 (37.3)	1578 (39.3)
Night	461 (11.7)	9 (15.3)	470 (11.7)
**Variable**	**Mean (SD)**	**Mean (SD)**	**Mean (SD)**
**Experience on current unit, y**	5.83 (5.93)	4.57 (3.83)	5.81 (5.91)
**Responsive behaviors by residents**	3.26 (1.66)	2.96 (1.73)	3.25 (1.66)
**Job satisfaction**	4.24 (0.64)	4.44 (0.51)	4.24 (0.64)
**Burnout**			
MBI Emotional Exhaustion	2.61 (1.68)	2.24 (1.69)	2.60 (1.68)
MBI Cynicism	2.68 (1.63)	2.77 (1.77)	2.68 (1.63)
MBI Efficacy	5.40 (0.85)	5.49 (0.70)	5.40 (0.85)
**Physical health**	49.1 (8.18)	50.0 (8.58)	49.1 (8.18)
**Mental health**	51.9 (8.48)	53.6 (8.05)	51.9 (8.48)

Abbreviations: MBI, Maslach Burnout Inventory; SD, standard deviation.

© 2020 Song Y et al. *JAMA Network Open.*

eTable 3. Multivariable Analyses of Organizational Context With Missed Care

	Logistic Component[a]			Poisson Component[b]		
**Variable**	**OR (95% CI)**	**Estimate**	***P* value**	**RR (95% CI)**	**Estimate**	***P* value**
Intercept	0.01 (0.003-0.04)	-4.52	<.001	3.31 (1.76-6.25)	1.20	<.001
**Organizational context**						
Less favorable	1 [Reference]	NA	NA	1 [Reference]	NA	NA
More favorable	1.59 (1.34-1.90)	0.47	<.001	0.92 (0.84-1.01)	-0.08	.08
**Age, y**						
<29	1 [Reference]	NA	NA	1 [Reference]	NA	NA
30–39	1.32 (1.00-1.74)	0.28	.05	0.88 (0.76-1.01)	-0.13	.06
40–49	1.32 (1.00-1.72)	0.27	.05	0.96 (0.84-1.09)	-0.04	.55
50–59	1.44 (1.08-1.91)	0.36	.01	1.03 (0.90-1.18)	0.03	.65
>60	1.52 (1.09-2.12)	0.42	.01	0.96 (0.81-1.14)	-0.04	.64
**Sex**						
Male	1 [Reference]	NA	NA	1 [Reference]	NA	NA
Female	0.84 (0.67-1.05)	-0.18	.12	1.12 (0.98-1.28)	0.11	.09
**Healthcare aide certificate received**						
No	1 [Reference]	NA	NA	1 [Reference]	NA	NA
Yes	1.32 (0.99-1.76)	0.28	.06	1.03 (0.87-1.22)	0.03	.71
**English as first language**						
Yes	1 [Reference]	NA	NA	1 [Reference]	NA	NA
No	1.57 (1.33-1.85)	0.45	<.001	1.06 (0.97-1.15)	0.06	.17
**Shift worked most of the time**						
Day	1 [Reference]	NA	NA	1 [Reference]	NA	NA
Evening	1.36 (1.18-1.58)	0.31	<.001	0.89 (0.82-0.97)	-0.11	.005
Night	2.95 (2.33-3.74)	1.08	<.001	1.1 (0.97-1.25)	0.09	.15
**Experience on current unit, y**	1 (0.99-1.01)	-0.002	.80	1.01 (1.00-1.02)	0.01	.005
**Responsive behaviors from residents**	0.87 (0.83-0.91)	-0.14	<.001	1.07 (1.04-1.09)	0.06	<.001
**Job satisfaction**	1.51 (1.33-1.72)	0.41	<.001	0.9 (0.85-0.95)	-0.11	<.001
**Burnout**						
MBI_Exhaustion	0.88 (0.83-0.94)	-0.13	<.001	1.05 (1.02-1.09)	0.05	.002
MBI_Cynicism	1.03 (0.98-1.09)	0.03	.22	1.03 (1.00-1.07)	0.03	.02
MBI_Efficacy	1.11 (1.02-1.21)	0.10	.02	0.95 (0.91-0.99)	-0.05	.01
**Physical health**	1.01 (1.00-1.02)	0.01	.26	1 (0.99-1.00)	-0.004	.12
**Mental health**	1.02 (1.01-1.03)	0.02	<.001	1 (1.00-1.01)	0.001	.67
**Unit type**						

© 2020 Song Y et al. *JAMA Network Open.*

	Logistic Component[a]			Poisson Component[b]		
**Variable**	**OR (95% CI)**	**Estimate**	***P* value**	**RR (95% CI)**	**Estimate**	***P* value**
General nursing home	1 [Reference]	NA	NA	1 [Reference]	NA	NA
Secure dementia	1.07 (0.84-1.36)	0.07	.58	1.04 (0.91-1.18)	0.04	.59
Non-secure dementia	1.31 (0.90-1.91)	0.27	.16	0.94 (0.75-1.18)	-0.06	.60
Secure mental health/psychiatric	2.27 (0.84-6.13)	0.82	.10	0.65 (0.27-1.58)	-0.43	.34
Other	0.76 (0.54-1.08)	-0.27	.12	0.9 (0.72-1.11)	-0.11	.31
**Unit staffing level**	1.09 (0.99-1.20)	0.08	.09	0.95 (0.90-1.01)	-0.05	.12
**Percent of care aide staffing hours out of total staffing**	1 (0.99-1.01)	-0.001	.81	1 (1.00-1.01)	0.003	.20
**Owner-operator model**						
Public not-for-profit	1 [Reference]	NA	NA	1 [Reference]	NA	NA
Private for-profit	1.08 (0.86-1.36)	0.08	.51	0.84 (0.74-0.96)	-0.17	.01
Voluntary not-for-profit	0.75 (0.59-0.96)	-0.28	.02	1.04 (0.91-1.18)	0.04	.60
**Nursing home size**						
Small	1 [Reference]	NA	NA	1 [Reference]	NA	NA
Medium	1.17 (0.90-1.53)	0.16	.24	0.93 (0.81-1.07)	-0.07	.32
Large	1.02 (0.79-1.31)	0.02	.90	0.79 (0.70-0.90)	-0.23	.001
**Health region**						
Winnipeg Regional Health Authority	1 [Reference]	NA	NA	1 [Reference]	NA	NA
Alberta Health Edmonton zone	1.41 (1.08-1.84)	0.34	.01	0.72 (0.62-0.84)	-0.32	<.001
Alberta Health Calgary zone	1.17 (0.89-1.54)	0.16	.25	0.83 (0.71-0.96)	-0.19	.02
BC Fraser Health Authority	0.67 (0.53-0.86)	-0.39	.002	0.82 (0.72-0.93)	-0.20	.003
BC Interior Health Authority	1.75 (1.14-2.68)	0.56	.01	0.79 (0.62-1.01)	-0.24	.06
**Random intercept for unit clustering**	1.08 (1.01-1.17)	0.08	.03	1.04 (1.01-1.06)	0.03	.001

Abbreviations: OR, odds ratio; NA, not applicable; RR, relative rate; MBI, Maslach Burnout Inventory; BC, British Columbia.
[a] Models the likelihood of 0 missed or rushed care.
[b] Compares numbers of missed or rushed care tasks among individuals with at least 1 missed or rushed care task.

© 2020 Song Y et al. *JAMA Network Open.*

eTable 4. Multivariable Analyses of Organizational Context With Rushed Care

	Logistic Component[a]			Poisson Component[b]		
**Variable**	**OR (95% CI)**	**Estimate**	***P* value**	**RR (95% CI)**	**Estimate**	***P* value**
Intercept	0 (<.001-0.01)	-6.54	<.001	7.46 (5.28-10.5)	2.01	<.001
**Organizational context**						
Less favorable	1 [Reference]	NA	NA	1 [Reference]	NA	NA
More favorable	1.66 (1.38-1.99)	0.50	<.001	0.93 (0.88-0.98)	-0.07	.007
**Age, y**						
<29	1 [Reference]	NA	NA	1 [Reference]	NA	NA
30–39	1.17 (0.86-1.58)	0.15	.33	1.05 (0.97-1.13)	0.05	.22
40–49	1.22 (0.90-1.63)	0.20	.20	1.04 (0.97-1.12)	0.04	.25
50–59	1.22 (0.89-1.66)	0.20	.21	1.03 (0.95-1.11)	0.03	.50
>60	1.21 (0.84-1.73)	0.19	.31	1.01 (0.92-1.10)	0.01	.90
**Sex**						
Male	1 [Reference]	NA	NA	1 [Reference]	NA	NA
Female	0.93 (0.73-1.17)	-0.08	.52	1.01 (0.95-1.08)	0.01	.71
**Healthcare aide certificate received**						
No	1 [Reference]	NA	NA	1 [Reference]	NA	NA
Yes	1.24 (0.91-1.68)	0.21	.17	1.04 (0.95-1.13)	0.03	.43
**English as first language**						
Yes	1 [Reference]	NA	NA	1 [Reference]	NA	NA
No	1.81 (1.51-2.18)	0.59	<.001	0.93 (0.89-0.97)	-0.07	.002
**Shift worked most of the time**						
Day	1 [Reference]	NA	NA	1 [Reference]	NA	NA
Evening	1.38 (1.17-1.61)	0.32	.0001	0.93 (0.89-0.97)	-0.07	.0009
Night	2.19 (1.72-2.77)	0.78	<.001	0.72 (0.66-0.78)	-0.33	<.001
**Experience on current unit, y**	0.99 (0.98-1.00)	-0.01	.20	1 (1.00-1.01)	0.003	.08
**Responsive behaviors from residents**	0.82 (0.79-0.86)	-0.19	<.001	1.05 (1.04-1.07)	0.05	<.001
**Job satisfaction**	1.86 (1.61-2.15)	0.62	<.001	0.88 (0.85-0.91)	-0.13	<.001
**Burnout**						
MBI_Exhaustion	0.82 (0.77-0.87)	-0.20	<.001	1.03 (1.02-1.05)	0.03	<.001
MBI_Cynicism	1.07 (1.01-1.13)	0.07	.02	0.99 (0.98-1.01)	-0.01	.34
MBI_Efficacy	1.15 (1.05-1.27)	0.14	.003	1 (0.97-1.02)	-0.004	.75
**Physical health**	1.02 (1.01-1.03)	0.02	.003	1 (1.00-1.00)	<.001	.88
**Mental health**	1.02 (1.01-1.03)	0.02	.001	1 (1.00-1.00)	-0.001	.23
**Unit type**						

© 2020 Song Y et al. *JAMA Network Open.*

	Logistic Component[a]			Poisson Component[b]		
**Variable**	**OR (95% CI)**	**Estimate**	***P* value**	**RR (95% CI)**	**Estimate**	***P* value**
General nursing home	1 [Reference]	NA	NA	1 [Reference]	NA	NA
Secure dementia	1.32 (1.03-1.70)	0.28	.03	1 (0.93-1.08)	0.003	.93
Non-secure dementia	1.21 (0.82-1.81)	0.19	.34	1.04 (0.94-1.16)	0.04	.45
Secure mental health/psychiatric	1.37 (0.58-3.24)	0.32	.47	0.9 (0.66-1.22)	-0.11	.49
Other	0.99 (0.69-1.41)	-0.01	.94	0.98 (0.89-1.09)	-0.02	.71
**Unit staffing level**	1.35 (1.22-1.50)	0.30	<.001	0.99 (0.95-1.02)	-0.01	.39
**Percent of care aide staffing hours out of total staffing**	0.99 (0.98-1.00)	-0.01	.02	1 (1.00-1.00)	0.001	.62
**Owner-operator model**						
Public not-for-profit	1 [Reference]	NA	NA	1 [Reference]	NA	NA
Private for-profit	1.24 (0.98-1.58)	0.22	.07	0.93 (0.87-0.99)	-0.07	.03
Voluntary not-for-profit	0.87 (0.68-1.12)	-0.13	.29	0.97 (0.91-1.04)	-0.03	.38
**Nursing home size**						
Small	1 [Reference]	NA	NA	1 [Reference]	NA	NA
Medium	1.24 (0.94-1.64)	0.22	.13	0.95 (0.88-1.02)	-0.05	.17
Large	1.22 (0.93-1.59)	0.20	.15	0.95 (0.88-1.02)	-0.06	.13
**Health region**						
Winnipeg Regional Health Authority	1 [Reference]	NA	NA	1 [Reference]	NA	NA
Alberta Health Edmonton zone	0.97 (0.74-1.28)	-0.03	.83	0.96 (0.88-1.03)	-0.05	.26
Alberta Health Calgary zone	0.97 (0.74-1.28)	-0.03	.84	0.9 (0.83-0.98)	-0.10	.01
BC Fraser Health Authority	0.9 (0.70-1.15)	-0.11	.39	0.94 (0.87-1.01)	-0.06	.09
BC Interior Health Authority	1.35 (0.86-2.12)	0.30	.19	0.9 (0.80-1.02)	-0.10	.09
**Random intercept for unit clustering**	1.06 (0.97-1.15)	0.06	.19	1.01 (1.00-1.02)	0.009	.009

Abbreviations: OR, odds ratio; NA, not applicable; RR, relative rate; MBI, Maslach Burnout Inventory; BC, British Columbia.
[a] Models the likelihood of 0 missed or rushed care.
[b] Compares numbers of missed or rushed care tasks among individuals with at least 1 missed or rushed care task.

© 2020 Song Y et al. *JAMA Network Open.*

eTable 5. Multivariable Analyses of 10 Elements of Organizational Context With Missed Care

	Logistic Component[a]			Poisson Component[b]		
**Variable**	**OR (95% CI)**	**Estimate**	***P* value**	**RR (95% CI)**	**Estimate**	***P* value**
Intercept	0.01 (0.00-0.06)	-5.13	<.0001	14.0 (4.10-48.0)	2.64	<.0001
**Organizational context element**						
ACT - Leadership	1.33 (0.86-2.05)	0.28	.20	0.84 (0.67-1.06)	-0.17	.15
ACT - Culture	0.38 (0.19-0.73)	-0.98	.004	1.51 (1.06-2.14)	0.41	.02
ACT - Evaluation	1.02 (0.64-1.65)	0.02	.92	1.07 (0.83-1.37)	0.06	.62
ACT - Formal interaction	0.90 (0.64-1.25)	-0.11	.52	1.08 (0.91-1.29)	0.08	.38
ACT - Informal interaction	1.09 (0.94-1.27)	0.09	.25	0.96 (0.88-1.05)	-0.04	.40
ACT - Social capital	1.06 (0.59-1.88)	0.06	.85	0.51 (0.36-0.70)	-0.68	<.0001
ACT - Structural and electronic resources	1.00 (0.84-1.18)	-0.004	.97	0.97 (0.88-1.07)	-0.03	.55
ACT - Organizational slack-staff	1.65 (1.32-2.05)	0.50	<.0001	0.80 (0.71-0.90)	-0.22	.0004
ACT - Organizational slack-space	0.98 (0.86-1.12)	-0.02	.78	1.01 (0.94-1.08)	0.01	.85
ACT - Organizational slack-time	2.03 (1.44-2.86)	0.71	<.0001	1.25 (1.03-1.50)	0.22	.02
**Age, y**						
<29	1 [Reference]	NA	NA	1 [Reference]	NA	NA
30–39	1.28 (0.97-1.68)	0.25	.08	0.88 (0.77-1.01)	-0.13	.06
40–49	1.30 (1.00-1.70)	0.26	.05	0.97 (0.85-1.10)	-0.03	.60
50–59	1.43 (1.08-1.89)	0.36	.01	1.03 (0.90-1.18)	0.03	.67
>60	1.51 (1.09-2.11)	0.41	.01	0.96 (0.81-1.13)	-0.04	.61
**Sex**						
Male	1 [Reference]	NA	NA	1 [Reference]	NA	NA
Female	0.85 (0.68-1.07)	-0.16	.16	1.11 (0.97-1.26)	0.10	.13
**Healthcare aide certificate received**						
No	1 [Reference]	NA	NA	1 [Reference]	NA	NA
Yes	1.26 (0.94-1.68)	0.23	.12	1.01 (0.86-1.20)	0.01	.87
**English as first language**						
Yes	1 [Reference]	NA	NA	1 [Reference]	NA	NA
No	1.48 (1.25-1.75)	0.39	<.0001	1.04 (0.96-1.14)	0.04	.34
**Shift worked most of the time**						
Day	1 [Reference]	NA	NA	1 [Reference]	NA	NA
Evening	1.39 (1.20-1.62)	0.33	<.0001	0.89 (0.82-0.96)	-0.12	.004
Night	3.00 (2.37-3.80)	1.10	<.0001	1.10 (0.97-1.25)	0.09	.15
**Experience on current unit, y**	1.00 (0.98-1.01)	-0.003	.69	1.01 (1.00-1.02)	0.01	.004
**Responsive behaviors from residents**	0.87 (0.83-0.91)	-0.14	<.0001	1.07 (1.04-1.09)	0.06	<.0001

© 2020 Song Y et al. *JAMA Network Open.*

	Logistic Component[a]			Poisson Component[b]		
Variable	OR (95% CI)	Estimate	*P* value	RR (95% CI)	Estimate	*P* value
**Job satisfaction**	1.47 (1.29-1.67)	0.38	<.0001	0.91 (0.86-0.97)	-0.09	.003
**Burnout**						
MBI_Exhaustion	0.89 (0.84-0.95)	-0.11	.0002	1.06 (1.02-1.09)	0.05	.0008
MBI_Cynicism	1.03 (0.97-1.08)	0.02	.36	1.03 (1.00-1.06)	0.03	.03
MBI_Efficacy	1.11 (1.02-1.21)	0.10	.02	0.95 (0.91-0.99)	-0.05	.02
**Physical health**	1.00 (1.00-1.01)	0.005	.34	1.00 (0.99-1.00)	-0.004	.14
**Mental health**	1.02 (1.01-1.03)	0.02	<.0001	1.00 (1.00-1.01)	0.0009	.69
**Unit type**						
General nursing home	1 [Reference]	NA	NA	1 [Reference]	NA	NA
Secure dementia	1.19 (0.95-1.49)	0.17	.13	1.02 (0.90-1.16)	0.02	.75
Non-secure dementia	1.08 (0.76-1.54)	0.08	.66	0.87 (0.36-2.09)	-0.14	.76
Secure mental health/psychiatric	1.61 (0.63-4.13)	0.47	.32	0.92 (0.75-1.13)	-0.09	.42
Other	0.84 (0.60-1.16)	-0.18	.29	0.99 (0.80-1.23)	-0.01	.96
**Unit staffing level**	0.97 (0.88-1.06)	-0.03	.48	0.96 (0.91-1.02)	-0.04	.20
**Percent of care aide staffing hours out of total staffing**	1.00 (0.99-1.01)	-0.0003	.93	1.00 (1.00-1.01)	0.002	.28
**Owner-operator model**						
Public not-for-profit	1 [Reference]	NA	NA	1 [Reference]	NA	NA
Private for-profit	0.93 (0.74-1.16)	-0.07	.52	0.87 (0.77-0.99)	-0.14	.04
Voluntary not-for-profit	0.74 (0.59-0.92)	-0.30	.007	1.08 (0.95-1.23)	0.08	.23
**Nursing home size**						
Small	1 [Reference]	NA	NA	1 [Reference]	NA	NA
Medium	1.13 (0.87-1.45)	0.12	.36	0.86 (0.75-0.98)	-0.15	.03
Large	1.05 (0.82-1.34)	0.05	.70	0.74 (0.65-0.84)	-0.31	<.0001
**Health region**						
Winnipeg Regional Health Authority	1 [Reference]	NA	NA	1 [Reference]	NA	NA
Alberta Health Edmonton zone	1.62 (1.26-2.09)	0.48	.0002	0.73 (0.63-0.84)	-0.32	<.0001
Alberta Health Calgary zone	1.04 (0.79-1.37)	0.04	.77	0.81 (0.69-0.95)	-0.21	.01
BC Fraser Health Authority	0.69 (0.55-0.88)	-0.37	.002	0.83 (0.73-0.94)	-0.19	.003
BC Interior Health Authority	1.56 (1.04-2.35)	0.45	.03	0.82 (0.64-1.04)	-0.20	.10
**Random intercept for unit clustering**	1.00 (0.95-1.06)	0.004	.88	1.02 (1.00-1.03)	0.02	.07

Abbreviations: ACT, the Alberta Context Tool; OR, odds ratio; NA, not applicable; RR, relative rate; MBI, Maslach Burnout Inventory; BC, British Columbia.

[a] Models the likelihood of 0 missed or rushed care.

[b] Compares numbers of missed or rushed care tasks among individuals with at least 1 missed or rushed care task.

© 2020 Song Y et al. *JAMA Network Open.*

eTable 6. Multivariable Analyses of 10 Elements of Organizational Context With Rushed Care

	Logistic Component[a]			Poisson Component[b]		
**Variable**	**OR (95% CI)**	**Estimat**	***P* value**	**RR (95% CI)**	**Estimate**	***P* value**
Intercept	0.86 (0.04-16.8)	-0.15	.92	1.26 (0.56-2.85)	0.23	.58
**Organizational context element**						
ACT - Leadership	0.75 (0.45-1.25)	-0.29	.26	0.93 (0.81-1.07)	-0.07	.31
ACT - Culture	0.70 (0.34-1.45)	-0.36	.33	1.34 (1.09-1.65)	0.29	.006
ACT - Evaluation	0.78 (0.46-1.30)	-0.25	.34	1.04 (0.89-1.21)	0.04	.63
ACT - Formal interaction	1.02 (0.71-1.46)	0.02	.91	1.06 (0.95-1.18)	0.06	.27
ACT - Informal interaction	0.90 (0.76-1.06)	-0.11	.20	1.03 (0.98-1.08)	0.03	.32
ACT - Social capital	0.67 (0.35-1.28)	-0.41	.22	1.24 (1.02-1.50)	0.21	.03
ACT - Structural and electronic resources	1.25 (1.03-1.52)	0.23	.02	0.94 (0.89-0.99)	-0.06	.03
ACT - Organizational slack-staff	1.91 (1.51-2.42)	0.65	<.0001	0.91 (0.85-0.98)	-0.09	.01
ACT - Organizational slack-space	0.95 (0.82-1.10)	-0.05	.50	1.00 (0.96-1.04)	-0.0009	.97
ACT - Organizational slack-time	1.14 (0.79-1.65)	0.13	.47	0.90 (0.80-1.01)	-0.11	.06
**Age, y**						
<29	1 [Reference]	NA	NA	1 [Reference]	NA	NA
30–39	0.98 (0.72-1.32)	-0.02	.89	1.07 (1.00-1.16)	0.07	.06
40–49	1.04 (0.78-1.40)	0.04	.77	1.06 (0.99-1.14)	0.06	.10
50–59	1.08 (0.80-1.46)	0.08	.63	1.04 (0.97-1.13)	0.04	.25
>60	1.01 (0.71-1.45)	0.01	.95	1.03 (0.94-1.13)	0.03	.53
**Sex**						
Male	1 [Reference]	NA	NA	1 [Reference]	NA	NA
Female	0.85 (0.67-1.07)	-0.16	.18	1.02 (0.95-1.09)	0.02	.54
**Healthcare aide certificate received**						
No	1 [Reference]	NA	NA	1 [Reference]	NA	NA
Yes	1.00 (0.74-1.34)	-0.004	.98	1.08 (0.99-1.18)	0.08	.10
**English as first language**						
Yes	1 [Reference]	NA	NA	1 [Reference]	NA	NA
No	1.76 (1.47-2.11)	0.57	<.0001	0.94 (0.90-0.99)	-0.06	.02
**Shift worked most of the time**						
Day	1 [Reference]	NA	NA	1 [Reference]	NA	NA
Evening	1.28 (1.09-1.50)	0.25	.003	0.93 (0.89-0.97)	-0.07	.0006
Night	1.81 (1.43-2.29)	0.59	<.0001	0.73 (0.67-0.79)	-0.32	<.0001
**Experience on current unit, y**	0.99 (0.98-1.01)	-0.01	.37	1.00 (1.00-1.01)	0.004	.02
**Responsive behaviors from residents**	0.82 (0.78-0.86)	-0.20	<.0001	1.05 (1.04-1.07)	0.05	<.0001

© 2020 Song Y et al. *JAMA Network Open.*

	Logistic Component[a]			Poisson Component[b]		
**Variable**	**OR (95% CI)**	**Estimat**	***P* value**	**RR (95% CI)**	**Estimate**	***P* value**
**Job satisfaction**	1.79 (1.55-2.08)	0.58	<.0001	0.86 (0.83-0.89)	-0.15	<.0001
**Burnout**						
MBI_Exhaustion	0.80 (0.75-0.85)	-0.23	<.0001	1.04 (1.02-1.06)	0.04	<.0001
MBI_Cynicism	1.06 (1.00-1.12)	0.06	.05	0.99 (0.97-1.01)	-0.01	.22
MBI_Efficacy	1.10 (1.01-1.21)	0.10	.04	1.01 (0.98-1.03)	0.01	.52
**Physical health**	1.01 (1.00-1.02)	0.01	.11	1.00 (1.00-1.00)	0.001	.32
**Mental health**	1.01 (1.00-1.02)	0.01	.12	1.00 (1.00-1.00)	-0.0002	.84
**Unit type**						
General nursing home	1 [Reference]	NA	NA	1 [Reference]	NA	NA
Secure dementia	1.22 (0.95-1.56)	0.20	.12	0.96 (0.89-1.04)	-0.04	.2935
Non-secure dementia	1.00 (0.69-1.46)	0.003	.99	1.09 (0.97-1.22)	0.08	.1343
Secure mental health/psychiatric	1.02 (0.43-2.46)	0.02	.96	0.93 (0.68-1.28)	-0.07	.6557
Other	1.01 (0.70-1.46)	0.01	.94	0.94 (0.85-1.05)	-0.06	.2709
**Unit staffing level**	1.18 (1.06-1.31)	0.16	.002	1.01 (0.97-1.04)	0.01	.77
**Percent of care aide staffing hours out of total staffing**	0.98 (0.97-0.99)	-0.02	<.0001	1.00 (1.00-1.00)	0.003	.03
**Owner-operator model**						
Public not-for-profit	1 [Reference]	NA	NA	1 [Reference]	NA	NA
Private for-profit	1.11 (0.87-1.42)	0.11	.40	0.98 (0.91-1.06)	-0.02	.66
Voluntary not-for-profit	0.84 (0.67-1.07)	-0.17	.15	0.97 (0.90-1.04)	-0.03	.35
**Nursing home size**						
Small	1 [Reference]	NA	NA	1 [Reference]	NA	NA
Medium	1.01 (0.77-1.33)	0.01	.93	0.98 (0.91-1.06)	-0.02	.66
Large	0.99 (0.76-1.28)	-0.01	.91	0.97 (0.90-1.05)	-0.03	.41
**Health region**						
Winnipeg Regional Health Authority	1 [Reference]	NA	NA	1 [Reference]	NA	NA
Alberta Health Edmonton zone	0.98 (0.74-1.28)	-0.02	.86	0.95 (0.87-1.03)	-0.05	.24
Alberta Health Calgary zone	1.09 (0.81-1.46)	0.08	.57	0.93 (0.85-1.02)	-0.07	.14
BC Fraser Health Authority	0.79 (0.62-1.02)	-0.23	.07	0.94 (0.87-1.02)	-0.06	.12
BC Interior Health Authority	1.03 (0.65-1.63)	0.03	.89	0.92 (0.81-1.05)	-0.08	.21
**Random intercept for unit clustering**	1.00 (0.90-1.12)	0.004	.94	1.01 (1.00-1.02)	0.01	.01

Abbreviations: ACT, the Alberta Context Tool; OR, odds ratio; NA, not applicable; RR, relative rate; MBI, Maslach Burnout Inventory; BC, British Columbia.

[a] Models the likelihood of 0 missed or rushed care.

[b] Compares numbers of missed or rushed care tasks among individuals with at least 1 missed or rushed care task.

© 2020 Song Y et al. *JAMA Network Open.*
